# Higher Serum Uric Acid Level Predicts Non-alcoholic Fatty Liver Disease: A 4-Year Prospective Cohort Study

**DOI:** 10.3389/fendo.2020.00179

**Published:** 2020-04-09

**Authors:** Fengjiang Wei, Jiaxin Li, Chen Chen, Kai Zhang, Li Cao, Ximo Wang, Jun Ma, Shuzhi Feng, Wei-Dong Li

**Affiliations:** ^1^Department of Genetics, College of Basic Medical Sciences, Tianjin Medical University, Tianjin, China; ^2^Tianjin Medical University, Tianjin, China; ^3^Tianjin Medical University General Hospital, Tianjin, China; ^4^Department of Health Statistics, College of Public Health, Tianjin Medical University, Tianjin, China

**Keywords:** non-alcoholic fatty liver disease, hyperuricemia, serum uric acid, risk factor, cohort study

## Abstract

**Background:** Non-alcoholic fatty liver disease (NAFLD) has become a serious disease affecting people's health in the world. This article studies the causal relationship between NAFLD and serum uric acid (SUA) levels.

**Methods:** During the 4 years of follow-up in a fixed cohort that was established in 2014, 2,832 follow-up subjects without NAFLD were finally included in this study. The study population was divided into four groups according to baseline SUA levels. Cox hazard regression model and Kaplan–Meier survival curves analysis were used to predict risk factors of NAFLD. The receiver operating characteristic curve analyses were used to determine SUA cutoffs for predicting NAFLD.

**Results:** The cumulative prevalence rates of NAFLD were 33.97% (962/2,832), 38.93% (758/1,947) in males and 23.05% (204/885) in females. The results showed that males had a higher incidence of NAFLD (χ^2^ = 68.412, *P* = 0.000). The Cox regression analysis disclosed that the hazard ratios of NAFLD [95% confidence interval (CI)] were 1.431 (95% CI, 1.123~1.823), 1.610 (95% CI, 1.262–2.054), and 1.666 (95% CI, 1.287–2.157) across the second to the fourth quartile of SUA adjusted for other confounders. The SUA cutoffs, sensitivity, specificity, and area under the curve (AUC) (95% CI) were ≥288.5 μmol/L, 75.5, 46.5%, 0.637(0.616–0.658), respectively, for total; ≥319.5 μmol/L, 65.8%, 48.4%, 0.590 (0.564–0.615), respectively, for males; and ≥287.5 μmol/L, 51.0%, 75.6%, 0.662 (0.619–0.704), respectively, for females. Kaplan–Meier survival curves revealed that individuals with higher SUA level had an increased risk of NAFLD in comparison to lower SUA level (*P* = 0.000).

**Conclusion:** Serum uric acid is positively correlated with NAFLD, and elevated SUA level can be used as an independent predictor for NAFLD.

## Introduction

Non-alcoholic fatty liver disease (NAFLD) is a very common chronic liver disease. The prevalence of NAFLD in the general population ranges from 20 to 30%, but its prevalence in the middle-aged population of Western countries can reach 46%, and 5 to 42% in Asian countries (1–6). As a component of metabolic syndrome (MetS), NAFLD is closely related to obesity, insulin resistance (IR), type 2 diabetes mellitus (T2DM), cardiovascular disease, and other chronic diseases (7, 8).

Serum uric acid (SUA) maintains balance in the body through a series of precise regulation mechanisms. Previously, numerous studies have suggested that the level of SUA will increase with the development of chronic metabolic diseases such as cardiovascular disease (9, 10), T2DM (11), and MetS (12–14). Studies in European or Korean populations have shown that SUA is associated with the occurrence and progression of NAFLD. A meta-analysis involving 55,573 participants indicated that the level of SUA was still related to NAFLD except for the confounding factors of sex, age, and MetS (15). A study that included 6,967 participants has the same conclusion (16). But in a cohort study involving 7,564 atomic bomb survivors, this association was found to be not statistically significant (17). In a cross-sectional study including 129 children and adolescents, the association between SUA and NAFLD was not observed (18). In a Chinese prospective cohort study (PMMJS) among 841 NAFLD males, they arrived to the conclusion that the level of SUA was negatively correlated with the remission rate of NAFLD (19). A cross-sectional study of 541 patients with type 2 diabetes showed that the association between SUA and NAFLD was found only in males, but not in females (20). The reason for different conclusions may be different sample size, population, definitions of hyperuricemia, lifestyle, and eating habits.

However, the causal relationship between hyperuricemia and NAFLD is controversial. Therefore, we established a prospective cohort study in the Chinese population to determine the causal relationship between SUA level and NAFLD. All subjects provided written informed consent prior to starting the study, and the protocol was approved by the Human Ethics Committee of Tianjin Medical University.

## Materials and Methods

### Participants

**Figure 1:**
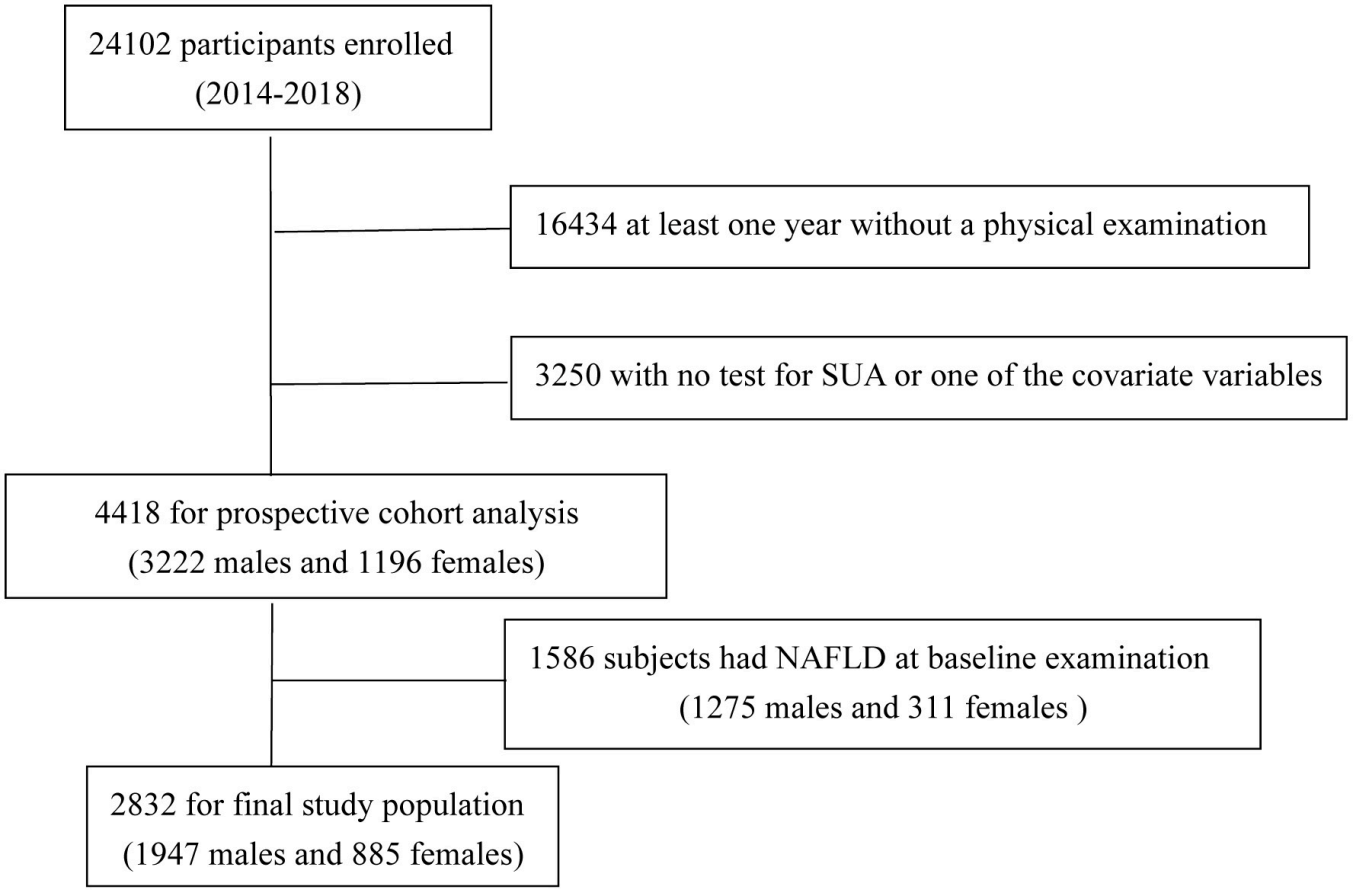
Flowchart of selection of study population.

We set up a follow-up cohort from April 2014 through October 2018. A total of 24,102 participants were enrolled in our cohort; after excluding the data that at least 1 year without a physical examination or with no test for SUA or one of the covariate variables, 4,418 subjects (3,222 men and 1,196 women) completed all inspections and measurements. Excluding 1,586 subjects (1,275 men and 311 women) with NAFLD at baseline examination, 2,832 subjects remained (1,947 men and 885 women, with an average age of 64.54 ± 14.66 years and 59.32 ± 15.83 years, respectively) in the study (Figure 1). Exclusion criteria were as follows: (1) already have NAFLD disease; (2) intake of alcohol >140 g/wk for males and 70 g/wk for females; (3) with a history of chronic liver disease.

### Ultrasonography

All subjects underwent hepatic ultrasonic examination by a trained sonographer who was not involved in the study. Ultrasonic instrument was a Toshiba Nemio 20 sonography machine (Toshiba, Tokyo, Japan) with a 3.5-MHz probe. All subjects were classified into two groups (with or without NAFLD) according to hepatic ultrasonic examination results.

### Measurement

All subjects underwent a yearly physical examination. Physiological index included height, weight, and blood pressure. Liver function index included total cholesterol (TC), high-density lipoprotein cholesterol (HDL), low-density lipoprotein cholesterol (LDL), and triglycerides (TGs); renal function index included SUA, serum creatinine (SCR), blood urea nitrogen (BUN), galactosyl glucosyltransferase, alanine aminotransferase (ALT), and aspartate transaminase. Glycometabolism index include glycated hemoglobin (HbA_1c_). Body mass index (BMI) was calculated (kg/m^2^) based on height and weight, and hypertension was defined as systolic blood pressure (SBP) of at least 140 mm Hg and/or diastolic blood pressure (DBP) of at least 90 mm Hg. The standard of hyperuricemia was SUA concentration ≥420 mol/L in men and ≥360 mol/L in women (21). The study population was divided into four groups according to the quartile of SUA levels. Estimated glomerular filtration rate (eGFR; mL/min per 1.73 m^2^) was calculated by using the equation proposed by investigators in the Chronic Kidney Disease Epidemiology Collaboration (22).

### Statistical Analysis

Anthropometric and biochemical features were categorized as continuous or categorical variables. Mean and standard deviation or the median and interquartile range were used for continuous variables statistical description. Comparisons between the two groups (with or without NAFLD) were performed using the Student *t*-test or Mann–Whitney *U*-test. Rate and ratio were used for dichotomous variables statistical description, whereas χ^2^ test was used for comparisons between groups. The multiple Cox regression was used to assess the relationship between SUA and NAFLD. The hazard ratios (HRs) and the corresponding 95% confidence intervals (95% CIs) for NAFLD were calculated after controlling for demographic and other comorbidities. The Kaplan–Meier survival curves were illustrated to reveal the risk of NAFLD in each SUA quartile, and non-parametric log-rank test was used for comparison among SUA quartiles. At the same time, the sensitivity, specificity, and areas under the receiver operating characteristic (ROC) curves (AUR) were calculated to evaluate the diagnostic effect of the SUA. Determination of diagnostic cutoff point value of SUA was identified by maximizing the Youden index (*J*) where *J* = (sensitivity + specificity) – 1 (23, 24). Cox regression analysis was used to analyze the influencing factors of NAFLD. Statistical analysis software was SPSS statistical software, version 19.0 (SPSS Inc., Chicago, IL, USA) for Windows, and significance level was set at α = 0.05.

## Results

### The Univariate Analyses of Characteristics

The univariate analyses of factors associated with NAFLD are shown in Table 1. There was no significant difference in mean age, eGFR, TBIL, DBIL, GLB, BUN, and TC between the two groups. Gender, BMI, SBP, DBP, SUA, ALT, TP, ALB, SCR, FBG (fasting plasma glucose), HbA_1c_, TGs, HDL-C, and LDL-C were associated with NAFLD in univariate analysis (*P* < 0.05). The NAFLD group had higher BMI, SBP, DBP, ALT, TP, ALB, SCR, TGs, FBG, LDL-C, and HbA_1c_ and lower HDL-C. The results showed that the NAFLD group had higher SUA level (Table 1).

**Table 1:** The univariate analyses of demographic and laboratory characteristics of patients with or without NAFLD.

**Variable**	**NAFLD**		***t*/χ^2^**	***P***
	**No**	**Yes**		
Gender (male/female)	1,189/681	758/204	68.412	0.000
Age (y)	63.02 ± 15.67	62.69 ± 14.34	0.555	0.579
BMI (kg/m^2^)	23.02 ± 2.69	25.37 ± 2.41	−22.571	0.000
eGFR (mL/min per 1.73 m^2^)	88.79 ± 18.51	89.16 ± 18.26	−0.510	0.610
SBP (mm Hg)	134.67 ± 20.95	138.81 ± 19.32	−5.085	0.000
DBP (mm Hg)	73.38 ± 11.70	76.48 ± 11.55	−6.664	0.000
SUA (μmol/L)	302.93 ± 77.09	338.88 ± 75.46	−11.839	0.000
ALT (IU/L)	14.87 ± 9.07	17.92 ± 11.74	−7.643	0.000
TBIL (μmol/L)	13.30 ± 5.43	13.58 ± 5.59	−1.294	0.196
DBIL (μmol/L)	4.73 ± 1.65	4.72 ± 1.63	0.243	0.808
TP (g/L)	73.08 ± 3.84	73.40 ± 3.91	−2.096	0.036
ALB (g/L)	46.24 ± 2.44	46.57 ± 2.33	−3.477	0.001
GLB (g/L)	26.84 ± 3.41	26.83 ± 3.49	0.074	0.941
BUN (mmol/L)	5.23 ± 1.45	5.28 ± 1.39	−0.847	0.397
SCR (μmol/L)	77.41 ± 17.74	79.90 ± 16.58	−3.615	0.000
FBG (mmol/L)	5.38 ± 1.04	5.53 ± 1.00	−3.735	0.000
HbA_1c_(%)	5.81 ± 0.68	5.89 ± 0.68	−2.951	0.003
TC (mmol/L)	5.00 ± 0.94	5.04 ± 0.94	−0.887	0.375
TGs (mmol/L)	1.12 ± 0.57	1.51 ± 0.97	−13.694	0.000
HDL-C (mmol/L)	1.52 ± 0.42	1.27 ± 0.32	15.855	0.000
LDL-C (mmol/L)	2.98 ± 0.83	3.10 ± 0.84	−3.532	0.000

*BMI, body mass index; eGFR, estimated glomerular filtration rate; SBP, systolic blood pressure; DBP, diastolic blood pressure; SUA, serum uric acid; ALT, alanine aminotransferase; TBIL, total bilirubin; DBIL, direct bilirubin; TP, plasma total protein; GLB, globulin; ALB, albumin; BUN, blood urea nitrogen; SCR, serum creatinine; FBG, fasting plasma glucose; HbA_1c_, glycosylated hemoglobin; TGs, plasma levels of triglycerides; TC, total cholesterol; HDL-C, high-density lipoprotein cholesterol; LDL-C, low-density lipoprotein cholesterol*.

### Association of SUA Level With Prevalence Rate of NAFLD

The cumulative prevalence rates of NAFLD after 4 years' follow-up was 33.97% (962/2,832): 38.93% (758/1,947) in males and 23.05% (204/885) in females, respectively (Table 2). Males had a higher incidence of NAFLD (χ^2^=49.860, *P* = 0.000). The prevalence rates of HUA (Hyperuricemia) identified by baseline SUA levels were 11.2% (318/2,832), 13.7% (266/1,947) in males, 6.1% (52/855) in females, respectively. The prevalence rates of HUA were higher in males (χ^2^=68.412, *P* = 0.000). The study population was divided into four quartiles by their SUA levels. As shown in Table 2, the overall prevalence of NAFLD was 18.51, 31.48, 40.06, and 45.62%, respectively, from the first quintile to the fourth in total. The results showed that the prevalence of NAFLD was significantly different among four quartiles (χ^2^=130.843, *P* = 0.000). The cumulative prevalence rates of NAFLD have a similar tendency in males and females. The results showed that SUA level was related to the prevalence of NAFLD.

**Table 2:** Association of SUA level with prevalence rate of NAFLD.

	**Quartiles**	**NAFLD**		**Total**	**Prevalence rates (%)**	**χ^2^**	***P***
		**No**	**Yes**				
Total	Quartile 1	568	129	697	18.51		
	Quartile 2	492	226	718	31.48		
	Quartile 3	425	284	709	40.06	130.843	0.000
	Quartile 4	385	323	708	45.62		
	Total	1,870	962	2,832	33.97		
MALES	Quartile 1	346	133	479	27.77		
	Quartile 2	310	184	494	37.25		
	Quartile 3	275	211	486	43.42	43.617	0.000
	Quartile 4	258	230	488	47.13		
	Total	1,189	758	1,947	38.93		
Females	Quartile 1	192	27	219	12.33		
	Quartile 2	182	40	222	18.02		
	Quartile 3	168	54	222	24.32	43.292	0.000
	Quartile 4	139	83	222	37.39		
	Total	681	204	885	23.05		

*Quartiles of SUA were defined as follows: first quartile, <258 μmol/L (< P_25_); second quartile, 258–310 μmol/L (P_25_~); third quartile, 310–362 μmol/L (P_50_~); and fourth quartile, ≥362 μmol/L (≥P_75_) (Total). Quartiles based on serum uric acid levels: first quartile, <288 μmol/L (< P_25_); second quartile, 288–333 μmol/L (P_25_~); third quartile, 333–383 μmol/L (P_50_~); and fourth quartile, ≥383 μmol/L (≥P_75_) (Males). Quartiles based on serum uric acid levels: first quartile, <222 μmol/L (< P_25_); second quartile, 222–257 μmol/L (P_25_~); third quartile, 257–301 μmol/L (P_50_~); and fourth quartile, ≥301 μmol/L (≥P_75_) (Females)*.

### The Cox Regression Analysis of SUA Levels for Incidence NAFLD

The Cox regression analysis was performed to evaluate association between SUA and NAFLD. Fourteen variables including gender, age, BMI, SBP, DBP, ALT, TP, SCR, ALB, FBG, HbA_1c_, TGs, HDL-C, and LDL-C were set as the independent variables, and with or without NAFLD as the dependent variable (Table 3). As shown in Table 3, among total study population, during the 4-year follow-up period from the index date, using the lowest SUA quintiles as reference, the crude HRs of NAFLD (95% CI) were 1.803 (95% CI, 1.453–2.239), 2.418 (95% CI, 1.964–2.978), and 2.864 (95% CI, 2.335–3.513) across the second to the fourth quartile of SUA in model 1 (unadjusted baseline values of variables); the HRs of NAFLD (95% CI) were 1.667 (95% CI, 1.342–2.094), 2.159 (95% CI, 1.729–2.696), and 2.487 (95% CI, 1.986–3.116) across the second to the fourth quartile of SUA in model 2 (model 1 adjusted for age and gender); the HRs of NAFLD (95% CI) were 1.431 (95% CI, 1.123–1.823), 1.610 (95% CI, 1.123–1.823), and 1.666 (95% CI, 1.287–2.157) across the second to the fourth quartile of SUA in model 3 (model 2 further adjusted for other confounders). The sex-specific association analysis between HUA and NAFLD has a similar tendency in females (Table 4, Models 4–6) and males (Table 5, Models 7–9). Notably, HUA was found to be an independent risk factor for NAFLD.

**Table 3:** The Cox regression analysis of SUA levels for incidence NAFLD during 4 years of follow-up among 2,832 subjects without NAFLD at the entry examination (total).

**Model**	***N***	**HR**	**95% CI**	***P***
**Model 1: unadjusted baseline values of variables**				
Quartile 1	697	—	—	0.000
Quartile 2^⋆^	718	1.803	1.453–2.239	0.006
Quartile 3^*^	709	2.418	1.964–2.978	0.000
Quartile 4^*^	708	2.864	2.335–3.513	0.000
SUA as a continuous variable (μmol/L)^*^	2,832	1.004	1.004–1.005	0.000
**Model 2: model 1 adjusted for age and gender**				
Quartile1	697	—	—	0.000
Quartile 2^*^	718	1.667	1.342–2.094	0.000
Quartile 3^*^	709	2.159	1.729–2.696	0.000
Quartile 4^*^	708	2.487	1.986–3.116	0.000
SUA as a continuous variable (μmol/L)^*^	2,832	1.004	1.003–1.005	0.000
**Model 3: model 2 further adjusted for other confounders**				
Quartile 1	697	—	—	0.000
Quartile 2^*^	718	1.431	1.123–1.823	0.004
Quartile 3^*^	709	1.610	1.262–2.054	0.000
Quartile 4^*^	708	1.666	1.287–2.157	0.000
SUA as a continuous variable (μmol/L)^*^	2,832	1.002	1.001–1.003	0.000

Model 1 (baseline): Quartiles based on SUA levels: first quartile, <258 μmol/L (< P_25_); second quartile, 258–310 μmol/L (P_25_~); third quartile, 310–362 μmol/L (P_50_~); and fourth quartile, ≥362μmol/L (≥P_75_). Model 2: model 1 adjusted for age and gender. Model 3: model 2 was further adjusted for BMI, SBP, DBP, ALT, TP, ALB, SCR, FBG, HbA_1c_, TGs, HDL-C, LDL-C. *P <0.05 was considered statistically significant.

**Table 4:** The Cox regression analysis of SUA levels for incidence NAFLD during 3 years of follow-up among 885 subjects without NAFLD at the entry examination (females).

**Model**	***N***	**HR**	**95% CI**	***P***
**Model 4: unadjusted baseline values of variables**				
Quartile 1	219	—	—	0.000
Quartile 2	222	1.502	0.922–2.447	0.103
Quartile 3^*^	222	2.091	1.317–3.319	0.002
Quartile 4^*^	222	3.459	2.240–5.341	0.000
SUA as a continuous variable (μmol/L)^*^	885	1.006	1.005–1.008	0.000
**Model 5: model 1 adjusted for age**				
Quartile 1	219	—	—	0.000
Quartile 2	222	1.435	0.880–2.340	0.148
Quartile 3^*^	222	1.933	1.215–3.076	0.005
Quartile 4^*^	222	3.001	1.929–4.670	0.000
SUA as a continuous variable (μmol/L)^*^	885	1.005	1.004–1.007	0.000
**Model 6: model 2 further adjusted for other confounders**				
Quartile 1	219	—	—	0.009
Quartile 2	222	1.425	0.803–2.529	0.226
Quartile 3^*^	222	1.954	1.150–3.322	0.013
Quartile 4^*^	222	2.495	1.481–4.202	0.001
SUA as a continuous variable (μmol/L)^*^	885	1.004	1.002–1.007	0.001

Model 4 (baseline): Quartiles based on SUA levels: first quartile, <222 μmol/L (< P_25_); second quartile, 222–257 μmol/L (P_25_~); third quartile, 257–301 μmol/L (P_50_~); and fourth quartile, ≥301 μmol/L (≥P_75_). Model 5: model 1 adjusted for age. Model 6: model 2 was further adjusted for BMI, SBP, DBP, ALT, TP, SCR, ALB, FBG, HbA_1c_, TGs, HDL-C, LDL-C. *P <0.05 was considered statistically significant.

**Table 5:** The Cox regression analysis of incidence NAFLD during 3 years of follow-up among 1,947 subjects without NAFLD at the entry examination (males).

**Model**	***N***	**HR**	**95% CI**	***P***
**Model 7: unadjusted baseline values of variables**				
Quartile 1	479	—	—	0.000
Quartile 2^*^	494	1.418	1.135–1.772	0.002
Quartile 3^*^	486	1.697	1.366–2.109	0.000
Quartile 4^*^	488	1.914	1.545–2.369	0.000
SUA as a continuous variable (μmol/L)^*^	1,947	1.003	1.002–1.004	0.000
**Model 8: model 1 adjusted for age**				
Quartile 1	479	—	—	0.000
Quartile 2^*^	494	1.385	1.108–1.732	0.004
Quartile 3^*^	486	1.657	1.333–2.059	0.000
Quartile 4^*^	488	1.899	1.534–2.352	0.000
SUA as a continuous variable (μmol/L)^*^	1,947	1.003	1.002–1.004	0.000
**Model 9: model 2 further adjusted for other confounders**				
Quartile 1	479	—	—	0.002
Quartile 2^*^	494	1.296	1.018–1.650	0.035
Quartile 3^*^	486	1.357	1.066–1.728	0.013
Quartile 4^*^	488	1.397	1.091–1.790	0.008
SUA as a continuous variable (μmol/L)^*^	1,947	1.001	1.001–1.003	0.040

Model 7 (baseline): Quartiles based on SUA levels: first quartile, <288 μmol/L (< P_25_); second quartile, 288–333 μmol/L (P_25_~); third quartile, 333–383 μmol/L (P_50_~); and fourth quartile, ≥383 μmol/L (≥P_75_). Model 8: model 1 adjusted for age. Model 9: model 2 was further adjusted for BMI, SBP, DBP, ALT, TP, SCR, ALB, FBG, HbA_1c_, TGs, HDL-C, LDL-C. *P <0.05 was considered statistically significant.

### Kaplan–Meier Survival Curves for NAFLD Among SUA Quartiles

**Figure 2:**
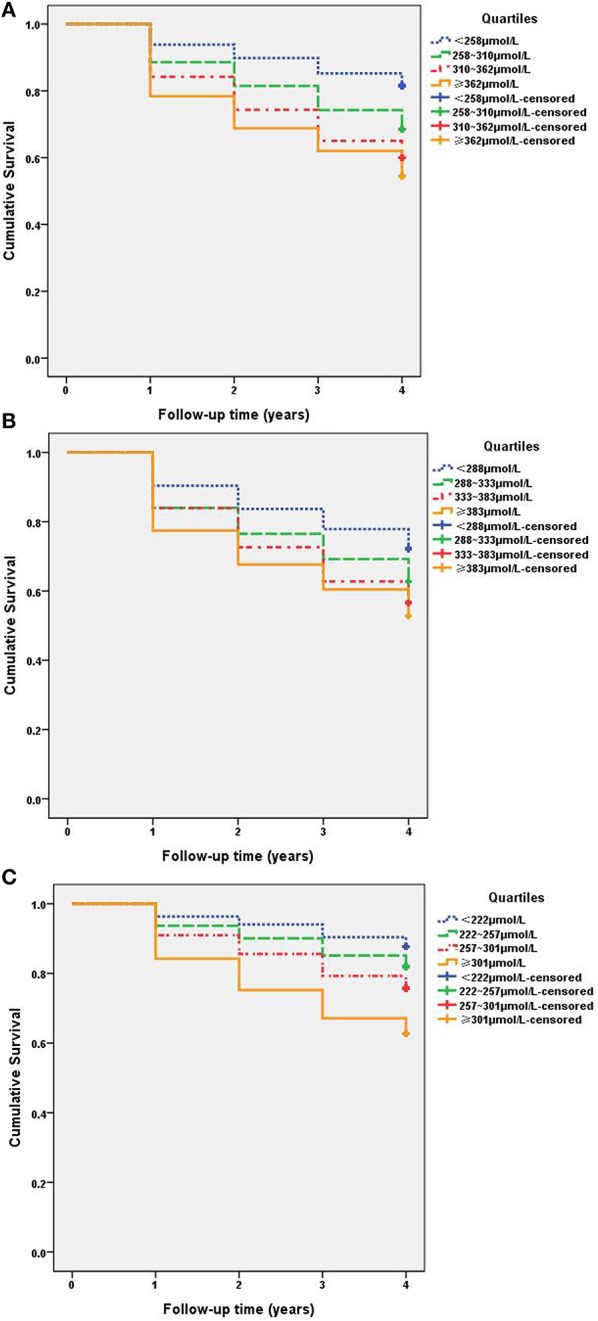
Kaplan–Meier survival curves for NAFLD among SUA quartiles. **(A)** Total: quartiles based on SUA levels: first quartile, <258 μmol/L (< *P*_25_); second quartile, 258–310 μmol/L (*P*_25_~); third quartile, 310–362 μmol/L (*P*_50_~); and fourth quartile, ≥362 μmol/L (≥*P*_75_). All subjects log-rank test *P* <0.05, < *P*_25_ vs. other quartiles. **(B)** Males: quartiles based on SUA levels: first quartile, <288 μmol/L (< *P*_25_); second quartile, 288–333 μmol/L (*P*_25_~); third quartile, 333–383 μmol/L (*P*_50_~); and fourth quartile, ≥383 μmol/L (≥*P*_75_). **(C)** Females: quartiles based on SUA levels: first quartile, <222 μmol/L (< *P*_25_); second quartile, 222–257 μmol/L (*P*_25_~); third quartile, 257–301 μmol/L (*P*_50_~); and fourth quartile, ≥301 μmol/L (≥*P*_75_).

As shown in Figure 2, Kaplan–Meier survival curves illustrate the differences in prevalence of NAFLD between different SUA quartiles. Among the total study population (Figure 2A), Kaplan–Meier survival curves revealed that individuals with higher SUA level had an increased risk of NAFLD in comparison to lower SUA level (*P* = 0.000). Similar results were found among males (Figure 2B) and females (Figure 2C). The log-rank test showed significance for all the SUA quartiles, the first quartile showed the lowest disease hazard for NAFLD, and the fourth quartile showed the highest disease hazard. The log-rank test showed that in the third quartile vs. the fourth quartile in total and males (Figures 2A,B), and the first quartile vs. the second quartile, as well as the second quartile vs. the third quartile in females (Figure 2C), there were no significant differences. However, other quartiles displayed significant difference. The above results showed that there is a dose–effect relationship between hyperuricemia and NAFLD; NAFLD onsets significantly changed with SUA quartiles.

### ROC Curve of the SUA Level as a Predictor of NAFLD

**Figure 3:**
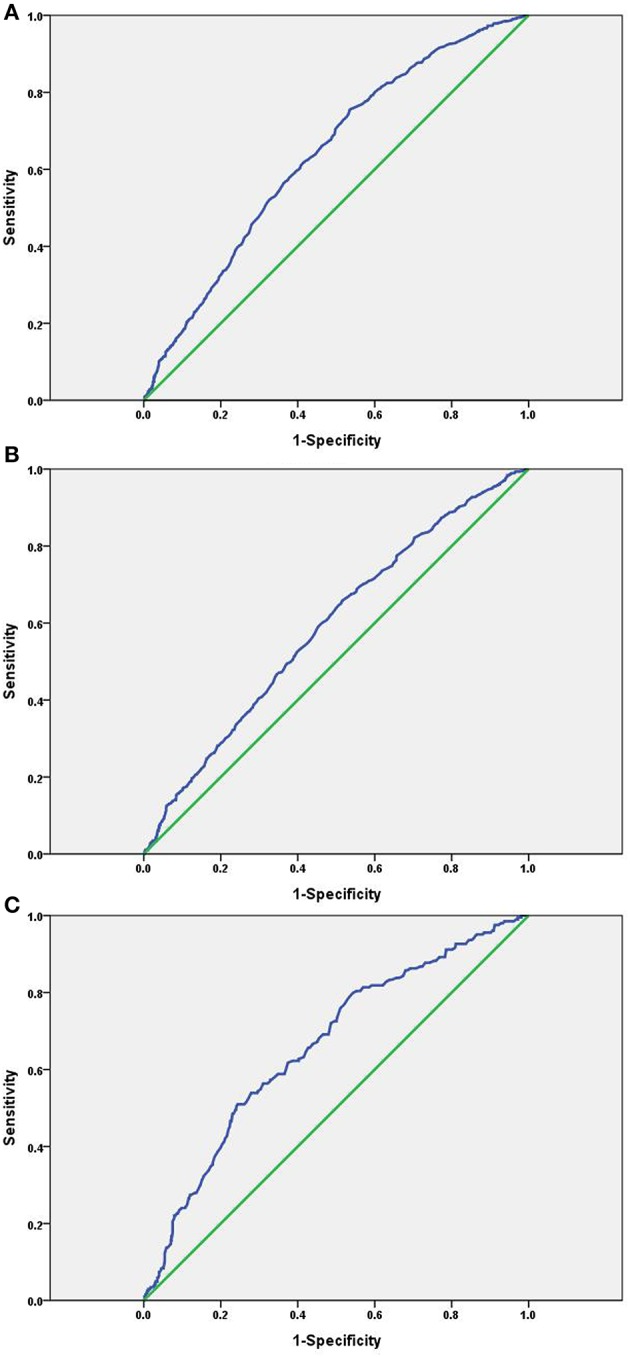
ROC curve of the SUA level as a predictor of NAFLD. **(A)** Total: AUC (95% CI) was 0.637(0.616–0.658); **(B)** males: AUC (95% CI) was 0.590 (0.564–0.615); **(C)** females: AUC (95% CI) was 0.662 (0.619–0.704).

Receiver operating characteristic analysis was used and calculated specificity and sensitivity of the prediction. The best cutoff value of SUA level to predict the incidence of NAFLD was ≥288.5 μmol/L, the AUC (95% CI) was 0.637 (0.616–0.658) with a sensitivity of 75.5% and specificity of 46.5% in total, as seen in Figure 3A. The best cutoff value was ≥319.5 μmol/L; the AUC (95% CI) was 0.590 (0.564–0.615) with a sensitivity of 65.8% and specificity of 48.4% in males, as seen in Figure 3B. The best cutoff value was ≥287.5 μmol/L; the AUC (95% CI) was 0.662 (0.619–0.704) with a sensitivity of 51.0% and specificity of 75.6% in females, as seen in Figure 3C.

## Discussion

Non-alcoholic fatty liver disease has a higher prevalence in the Western world, and which is becoming an emerging health threat in Asia (25). We prospectively followed 2,832 subjects who were free from NAFLD at baseline examination from April 2014 through October 2018. Our results show that SUA level can be used as an independent predictor of NAFLD in a fixed cohort Chinese population.

In cross-sectional and prospective studies, SUA level was a risk factor for NAFLD. In a study including 242 male patients with NAFLD [102 with non-alcoholic steatohepatitis (NASH) and 140 with simple steatosis (SS)], the study found that SUA was associated with early liver damage in patients with NAFLD, and SUA levels were significantly higher in subjects with NASH than those of SS (26). More recently, a cross-sectional and longitudinal population study showed that SUA is related to the occurrence and development of NAFLD. Additionally, the pathogenic effect of SUA levels on fatty liver is more significant in female population than in males (27). In a meta-analysis of 11 studies that were done in various countries, including China, Korea, Japan, India, and United States, they found a significant association between SUA and NAFLD. The risk of NAFLD was increased almost 2-fold in the highest SUA group compared to the lowest group (28). As expected, we performed the Cox regression analysis, and our results showed that the HRs of NAFLD (95% CI) were 1.431 (95% CI, 1.123–1.823), 1.610 (95% CI, 1.262–2.054), and 1.666 (95% CI, 1.287–2.157) across the second to the fourth quartile of SUA vs. the first quartile after adjusting for other confounders. The sex-specific association analysis between HUA and NAFLD has a similar tendency in males and females. Our findings suggest that elevated SUA levels promote the development of NAFLD, and which is consistent with the previous hypothesis that SUA might be an important contributor to the development of NAFLD.

In our studies, the Kaplan–Meier survival curves revealed that individuals with higher SUA level had an increased risk of NAFLD in comparison to lower SUA level (*P* < 0.001). HUA predicted higher incidences of NAFLD in a dose-dependent manner; NAFLD onsets significantly differed across SUA quartiles. Our results are consistent with other studies conducted on Chinese population. In study of two distinct ethnic groups, Uyghur and Han in northwest China, the major findings were that SUA concentrations and NAFLD were correlated in both populations, but Uyghurs had a higher prevalence of NAFLD. This finding may indicate that some related factors such as lifestyle, dietary habits, and genetic susceptibility play a more important role in the pathogenesis of NAFLD in Uyghurs than in Hans (29). In a Chinese cross-sectional study including 8,925 subjects, the results also demonstrated the effect of SUA on NAFLD (30). In the Cardiometabolic Risk in Chinese study, they found strong positive associations between elevated SUA levels and NAFLD risk in the non-hypertensive Chinese adults, independent of other metabolic changes (31). Another prospective study that followed 6,890 men and women found a positive correlation between SUA and NAFLD (32).

The pathogenesis of NAFLD is very complex, and its specific causes are not fully explained. The occurrence and development of NAFLD are the result of genetic and environmental factors. Several major hexose–uric acid transporters, including SLC2A9 and ABCG2, are highly expressed in the liver and kidney (33, 34). In our previous studies, we found that the plasma uric acid level was dynamically coupled with the HbA_1c_ level, depending on different stages of normal, impaired glucose tolerance, and diabetes. It seems uric acid is a regulator, or at least regulated, by the plasma glucose level (35). The first pathogenic mechanism was metabolic disturbances. Oxidative stress and lipid peroxidation were the main causes of fatty liver (36), whereas SUA was the main antioxidant *in vivo*, which was significantly associated with the degree of steatosis and the greater odds of advanced lobular inflammation of NAFLD (37). Another pathogenic mechanism was IR. Hyperuricemia is a component of MetS; the increase in SUA level could promote oxidative stress and reactive oxygen species level. Because of the above reasons, it would cause IR and abnormal blood glucose metabolism in the body and then cause the occurrence of NAFLD (38). Lanaspa et al. (39) found that uric acid can directly stimulate hepatic fat accumulation. This is a good supplement to the pathogenesis of NAFLD caused by SUA.

## Conclusion

In summary, we conducted a follow-up study in the Chinese population, and the results showed that after excluding other confounding factors there was a causal relationship between SUA level and NAFLD. Serum uric acid can be used as an independent predictor of NAFLD. There was a dose–effect relationship between hyperuricemia and NAFLD; NAFLD onsets significantly changed with SUA quartiles. Further studies on the mechanism of NAFLD caused by SUA will not only broaden our comprehension of the NAFLD mechanisms, but also assist in the eventual development of new prevention and treatment strategies for the NAFLD.

## Data Availability Statement

The datasets generated for this study are available on request to the corresponding author.

## Ethics Statement

The studies involving human participants were reviewed and approved by Human Ethics Committee of Tianjin Medical University. The patients/participants provided their written informed consent to participate in this study.

## Author Contributions

W-DL conceived and designed the study. W-DL and FW wrote the manuscript. JM, SF, FW, CC, KZ, LC, JL, and XW collected subjects and clinical data. FW and JL analyzed the data. All authors have reviewed the manuscript.

### Conflict of Interest

The authors declare that the research was conducted in the absence of any commercial or financial relationships that could be construed as a potential conflict of interest.
